# Engineering low-endotoxin lipid A in a double auxotroph *Pseudomonas aeruginosa* to develop safer whole-cell vaccines

**DOI:** 10.3389/fcimb.2026.1840122

**Published:** 2026-06-01

**Authors:** Víctor Fuentes-Valverde, Patricia García, Ana Candela, Rebeca Santamarina-Fernández, José Avendaño-Ortiz, Emma Martínez-Alonso, Marina Oviaño, Rafael Cantón, Jesús Arenas, Miriam Moscoso, Germán Bou

**Affiliations:** 1 Servicio de Microbiología Clínica and Grupo de Investigación en Microbiología, Instituto de Investigación Biomédica de A Coruña (INIBIC), Complexo Hospitalario Universitario de A Coruña (CHUAC), SERGAS, Universidade da Coruña, A Coruña, Spain; 2 Centro de Investigación Biomédica en Red de Enfermedades Infecciosas (CIBERINFEC), Instituto de Salud Carlos III, Madrid, Spain; 3 Servicio de Microbiología, Hospital Universitario Ramón y Cajal and Instituto Ramón y Cajal de Investigación Sanitaria (IRYCIS), Madrid, Spain; 4 Department of Research, Hospital Universitario Ramón y Cajal, Instituto Ramón y Cajal de Investigación Sanitaria (IRYCIS), Madrid, Spain; 5 Proteomics Unit, Hospital Universitario Ramón y Cajal, Instituto Ramón y Cajal de Investigación Sanitaria (IRYCIS), Madrid, Spain; 6 Unit of Microbiology and Immunology, Faculty of Veterinary, University of Zaragoza, Agroalimentary Institute of Aragon, Zaragoza, Spain; 7 Departamento de Fisioterapia, Medicina e Ciencias Biomédicas, Universidade da Coruña, A Coruña, Spain

**Keywords:** auxotrophy, humoral immunity, lipopolysaccharide, live-attenuated vaccines, protective efficacy, *Pseudomonas aeruginosa*

## Abstract

**Introduction:**

*Pseudomonas aeruginosa* is a highly adaptable Gram-negative bacterium causing severe respiratory infections, particularly in vulnerable populations. The rise of antimicrobial resistance highlights the urgent need for effective vaccines. We previously developed a live-attenuated vaccine candidate, PAO1 Δ*murI* Δ*alr* Δ*dadX*, a genetically stable double auxotrophic strain that exhibited dose-dependent reactogenicity upon intranasal administration in mice, likely due to lipid A component of lipopolysaccharide (LPS).

**Methods:**

To reduce LPS-associated toxicity while preserving immunogenicity, we engineered novel strains by inactivating genes involved in lipid A biosynthesis (*htrB1*, *htrB2*) or modification (*pagP*, *pagL*). Lipid A structural modifications were confirmed by MALDI-TOF MS. Mutants were evaluated for Toll-like receptor 4 (TLR4) activation, virulence attenuation, and their ability to induce immune responses and protection in an acute pneumonia model.

**Results:**

All engineered strains displayed modified lipid A structures. Mutations in *htrB1* and *htrB2* reduced TLR4 activation and significantly attenuated virulence following intraperitoneal challenge in mice. Inactivation of *pagL* resulted in minimal attenuation, whereas *pagP* inactivation led to marked attenuation without altering TLR4 activation. In the acute pneumonia model, all mutants elicited robust systemic and mucosal immune responses, and conferred strong protection, despite transient weight loss following intranasal administration.

**Discussion:**

Targeted lipid A modification represents an effective strategy to reduce *in vitro* reactogenicity while preserving immunogenicity in live-attenuated *P. aeruginosa* vaccine candidates. Although further optimization may minimize residual *in vivo* effects, these findings support the potential of engineered strains as vaccine candidates for preventing respiratory infections caused by *P. aeruginosa*.

## 1 Introduction

*Pseudomonas aeruginosa* is a Gram-negative opportunistic pathogen with a highly versatile metabolism that causes a wide range of diseases, particularly in immunocompromised individuals, people with cystic fibrosis (pwCF), and patients with skin or mucosal injuries (Killough et al., 2022). In 2019, *P. aeruginosa* ranked as the third leading cause of ventilator-associated pneumonia in intensive care units (ECDC, 2023). Treatment is challenging due to its ability to develop resistance through intrinsic, acquired, and adaptive mechanisms (Botelho et al., 2019; Qin et al., 2022). In response to the rising threat of antimicrobial resistance, vaccines have emerged as a promising alternative for combating this pathogen (Killough et al., 2022). Among the various experimental live-attenuated vaccines developed to date, the candidate PAO1 Δ*murI*, a D-glutamate auxotroph (Cabral et al., 2017), and its derivative PAO1 Δ*murI* Δ*alr* Δ*dadX*, hereafter referred to as PAO1 ΔΔΔ, which is auxotrophic for D-glutamate and D-alanine (Fuentes-Valverde et al., 2022), have demonstrated efficacy when administered intranasally (IN). Both strains elicit robust systemic and mucosal antibody responses and significantly improve survival in murine models of acute pulmonary infections with cytotoxin-producing *P. aeruginosa* strains (Cabral et al., 2020; Fuentes-Valverde et al., 2022). Notably, PAO1 ΔΔΔ offers the additional benefit of enhanced genetic stability over its parent strain, which is desirable for biosafety. However, high IN doses have resulted in adverse effects in mice, likely due to the endotoxin activity of lipopolysaccharide (LPS) (Cabral et al., 2020; Fuentes-Valverde et al., 2022; Moscoso et al., 2025).

LPS is the main structural component of the outer membrane (OM) of *P. aeruginosa* and a major virulence factor in respiratory infections (Pier, 2007). LPS is composed of three elements: lipid A, a core oligosaccharide, and the O-antigen. It acts as a physical barrier, induces reactive oxygen species production, triggers inflammation, and contributes to antibiotic resistance (Huszczynski et al., 2020). Lipid A is essential for bacterial survival and anchors LPS to the OM. The canonical LPS structure is based on *Escherichia coli*, whose biosynthesis occurs at the cytosolic interface of the inner membrane via the Raetz pathway (Whitfield and Trent, 2014). Lipid A comprises a glucosamine disaccharide linked by a β(1’,6) glycosidic bond, with phosphate groups at the 1 and 4’ positions. Fatty acids are attached at positions 2 and 2’ via amide bonds, and at positions 3 and 3’ linked via ester bonds, forming the tetra-acylated lipid IV_A_ precursor. Secondary acyl chains are added by LpxL (known as HtrB1/HtrB2 in *P. aeruginosa*) and by LpxM at the 2 and 2’-positions, respectively (Moskowitz and Ernst, 2010; Hittle et al., 2015), yielding the hexa-acylated lipid A. The lipid A disaccharide is linked at the 6’ position to the core oligosaccharide through one or more 3-deoxy-D-manno-octulosonic acid (Kdo) residues (Whitfield and Trent, 2014), and translocated to the periplasm, where the O-antigen is incorporated before transport of the mature LPS to the OM (Whitfield and Trent, 2014).

The penta-acylated lipid A of *P. aeruginosa* displays a bis-phosphorylated structure, consisting of a β(1,6)-linked diglucosamine backbone with primary fatty acids [one 3-hydroxydecanoic acid (3OH-C10) and two 3-hydroxydodecanoic acid (3OH-C12)], secondary acyl chains with distinct hydroxylation patterns (2OH-C12 and a C12 chain) (Hofstaedter et al., 2024a), and two phosphate groups located at the 1 and 4’ positions of the glucosamine disaccharide (**Supplementary Figure 1A**). However, in isolates from pwCF, a hexa-acylated lipid A variant is often produced due to the addition of palmitate (C16) by the enzyme PagP that acylates the 3’ position, unlike in Enterobacterales, where it modifies the 2 position (Ernst et al., 1999; Bishop et al., 2000; Thaipisuttikul et al., 2014). The constitutive expression of palmitoylated lipid A in CF strains suggests a role in bacterial adaptation and survival in the lungs (Moskowitz and Ernst, 2010). This modification increases resistance to cationic antimicrobial peptides and triggers a stronger pro-inflammatory response. Additionally, some CF isolates produce a hepta-acylated lipid A variant, retaining 3OH-C10 at position 3, likely due to the inactivation of the acyltransferase PagL (Geurtsen et al., 2005; Cigana et al., 2009). PagL hydrolyzes the ester bond at position 3 of lipid A, removing the 3OH-C10 acyl chain in *P. aeruginosa*; however, when expressed in *E. coli*, this enzyme shows no preference for acyl chain length (3OH-C10 or 3OH-C14) (**Supplementary Figure 1B**) (Trent, 2004; Geurtsen et al., 2005). This modification of lipid A has been associated with increased resistance to β-lactam antibiotics and reduced stimulation of proinflammatory interleukins, with no effect on aminoglycoside susceptibility (Kawasaki et al., 2004; Ernst et al., 2006; Hofstaedter et al., 2024b). Moreover, *P. aeruginosa* lipid A can be modified by two distinct dioxygenases, LpxO1 and LpxO2, which catalyze the site-specific hydroxylation of secondary acyl chains. These enzymes act in coordination with the late acyltransferases: LpxO1 hydroxylates the 2’-acyloxyacyl laurate added by HtrB2, whereas LpxO2 targets the 2-acyloxyacyl laurate incorporated by HtrB1 (Hofstaedter et al., 2024a). Although these enzymes are not part of the canonical Raetz biosynthetic pathway, lipid A modifications occur post-synthetically, and contributes to the structural diversification of *Pseudomonas* spp. lipid A and its adaptation to environmental conditions.

The lipid A-modifying enzymes are tightly regulated, mainly by the two-component signal transduction systems PmrAB and PhoPQ (Needham et al., 2013). PmrAB responds to Fe³^+^ or acidic pH (Wosten et al., 2000) and regulates the addition of 4-amino-4-deoxy-L-arabinose (Ara4N) and phosphoethanolamine (pEtN) to lipid A, reducing the negative charge on the bacterial cell surface and increasing resistance to cationic antimicrobial peptides (Lee et al., 2004; Moskowitz et al., 2004). PmrAB is further regulated post-transcriptionally by the PhoPQ system through the protein PmrD (Kox et al., 2000; Gunn, 2001). PhoPQ responds to low Mg²^+^ and directly regulates the expression of several lipid A-modifying enzymes such as PagP, PagL, and LpxO1, and LpxO2 (Bishop et al., 2000; Castelli et al., 2000; Murata et al., 2007; MacArthur et al., 2011). These lipid A modifications affect resistance to polymyxins and host antimicrobial peptides and modulate the inflammatory response (Ernst et al., 2003; Chilton et al., 2012; Maeshima and Fernandez, 2013; Mazgaeen and Gurung, 2020).

LPS is the primary activator of the inflammatory response to Gram-negative bacteria (Mazgaeen and Gurung, 2020). Upon release, the LPS monomer delivers lipid A to the Toll like receptor 4 (TLR4)/myeloid differentiation factor 2 (MD-2) complex through two accessory proteins, LPS-binding protein and CD14, promoting receptor dimerization and intracellular signaling (Park et al., 2009). TLR4/MD-2 activation triggers two signaling pathways: the MyD88-dependent pathway, inducing pro-inflammatory cytokines, and the TRIF-mediated pathway, which triggers cytokines with a reduced inflammatory profile (Simpson and Trent, 2019). Immunological activity depends on the number of acyl chains: hexa-acylated lipid A is approximately 100 times more active than the penta-acylated forms (Park et al., 2009; SenGupta et al., 2016). Modifications to the phosphate groups of lipid A, such as removing one phosphate or adding Ara4N, reduce the endotoxin activity by disrupting hydrophilic interactions (Park et al., 2009).

Our study aims to reduce the endotoxicity of the vaccine candidate PAO1 ΔΔΔ by modifying the structure of its lipid A without compromising its immunogenicity or protective efficacy. To achieve this, genes encoding enzymes involved in lipid A biosynthesis (HtrB1 and HtrB2), as well as those involved in the LPS modification pathway, including PagP and PagL, were selectively inactivated.

## 2 Materials and methods

### 2.1 Bacterial strains, growth conditions and plasmids

The bacterial strains and plasmids used in this study are listed in **Table 1**. All strains were cultured in LB (Lysogeny Broth/Luria-Bertani) broth or on LB medium with 2% agar with aeration (180 rpm) at 37°C. *P. aeruginosa* PAO1 Δ*murI* was grown in LB medium supplemented with 10 mM D-glutamate (Sigma-Aldrich, Inc.), while PAO1 ΔΔΔ and its mutant derivatives were cultured in LB supplemented with 8 mM D-glutamate and 6 mM D-alanine (Sigma-Aldrich, Inc.), unless otherwise indicated. Growth and viability of PAO1 ΔΔΔ and its mutant derivatives were assessed by inoculating single colonies into supplemented LB and incubating at 37 °C with shaking for 16 h. Cultures were diluted to an initial optical density at 600 nm (OD_600_) of 0.01 in fresh LB (with or without the D-amino acids) and grown for 8 h. Samples were taken every hour to measure culture turbidity (OD_600_) and determine colony-forming units (CFU) on supplemented LB agar. All assays were performed in triplicate.

**Table 1:** Bacterial strains and plasmids used in this study.

Strain or plasmid	Relevant features	Reference
*P. aeruginosa* strains		
PAO1	Reference strain. Isolate from a patient with a burn wound infection.	CECT 4122; (Stover et al., 2000)
PAO1 Δ*murI*	PAO1 derivative lacking *murI* gene (Locus tag PA4662). Auxotroph for D-glutamate.	(Cabral et al., 2017)
PAO1 Δ*murI* Δ*alr*	PAO1 Δ*murI* derivative lacking *alr* gene (Locus tag PA4930) (Δ*alr*).	(Fuentes-Valverde et al., 2022)
PAO1 Δ*murI* Δ*alr* Δ*dadX* (= PAO1 ΔΔΔ)	PAO1 Δ*murI* Δ*alr* derivative lacking *dadX* gene (Locus tag PA5302). Auxotroph for D-glutamate and D-alanine.	(Fuentes-Valverde et al., 2022)
PAO1 ΔΔΔ Δ*htrB1*	PAO1 ΔΔΔ derivative lacking *htrB1* gene (Locus tag PA0011; homologous to *E. coli lpxL2*).	This study and (Hernández-García et al., 2024)
PAO1 ΔΔΔ Δ*htrB2*	PAO1 ΔΔΔ derivative lacking *htrB2* gene (Locus tag PA3242; homologous to *E. coli lpxL1*).	This study and (Hernández-García et al., 2024)
PAO1 ΔΔΔ Δ*pagP*	PAO1 ΔΔΔ derivative lacking *pagP* gene (Locus tag PA1343).	This study
PAO1 ΔΔΔ Δ*pagL*	PAO1 ΔΔΔ derivative lacking *pagL* gene (Locus tag PA4661).	This study
PAO1 ΔΔΔ Δ*htrB1* Δ*pagP*	PAO1 ΔΔΔ Δ*htrB1* derivative lacking *pagP* gene (Locus tag PA1343).	This study
PAO1 ΔΔΔ Δ*htrB2* Δ*pagP*	PAO1 ΔΔΔ Δ*htrB2* derivative lacking *pagP* gene (Locus tag PA1343).	This study
PA14	Hypervirulent strain isolated from a patient with a burn wound infection.	(Lee et al., 2006)
*E. coli* strains		
S17-1 (λ*pir*)	*recA*^–^, *thi*^–^, *pro*^–^, *hsd*R^–^ (RP4-2-Tc::Mu Km::Tn7 λ*pir^+^*)	(Simon et al., 1983)
SM10 (λ*pir*)	*thi-1 thr leu tonA lacY supE recA*::RP4-2-Tc::Mu, KmR, RP4+ (λ*pir+*)	(Herrero et al., 1990). Kindly provided by Dr. E. Díaz (CIB-CSIC, Madrid)
BL21	*fhuA2* (*lon*) *ompT gal* (*dcm*) Δ*hsdS*	Lab collection (Studier and Moffatt, 1986)
Plasmids		
pEX18Gm	Suicide vector. *oriT^+^ sacB^+^.* Gm^R^.	(Hoang et al., 1998)
pKNG101	Suicide vector. *oriR6K^+^*, *sacB^+^*, *mobRK2*, Sm^R^, Amp^R^.	(Kaniga et al., 1991). Kindly provided by Dr. M.T. Zamarro (CIB-CSIC, Madrid)
pKNG101(UP-Δ*alr*-DN)	pKNG101 derivative containing the 5’ and 3’ flanking regions of the *alr* (PA4930) gene of *P. aeruginosa* PAO1.	(Fuentes-Valverde et al., 2022)
pKNG101(UP-Δ*dadX*-DN)	pKNG101 derivative containing the 5’ and 3’ flanking regions of the *dadX* (PA5302) gene of *P. aeruginosa* PAO1.	(Fuentes-Valverde et al., 2022)
pKNG101(UP-Δ*htrB1*-DN)	pKNG101 derivative containing the 5’ and 3’ flanking regions of the *htrB1* gene (PA0011) of *P. aeruginosa* PAO1.	This study
pKNG101(UP-Δ*htrB2*-DN)	pKNG101 derivative containing the 5’ and 3’ flanking regions of the *htrB2* gene (PA3242) of *P. aeruginosa* PAO1.	This study
pEX18Gm(UP-Δ*pagP*-DN)	pEX18Gm derivative containing the 5’ and 3’ flanking regions of the *pagP* (PA1343) gene of *P. aeruginosa* PAO1.	This study
pKNG101(UP-Δ*pagL*-DN)	pKNG101 derivative containing the 5’ and 3’ flanking regions of the *pagL* (PA4661) gene of *P. aeruginosa* PAO1.	This study

CECT, Spanish Type Culture Collection; R, Resistance to antimicrobials; Km, kanamycin; INIBIC-CHUAC: Instituto de Investigación Biomédica de A Coruña - Complejo Hospitalario Universitario A Coruña; CIB-CSIC: Centro de Investigaciones Biológicas - Consejo Superior de Investigaciones Científicas.

For lipid A analysis under magnesium-limiting conditions, bacteria were grown in N minimal medium [5 mM KCl, 7.5 mM (NH_4_)_2_SO_4_, 0.5 mM K_2_SO_4_, 1 mM KH_2_PO_4_, 0.1 M Tris-HCl, 0.1% w/v casamino acids, and 38 mM glycerol], supplemented with either 8 µM (inducing conditions) or 1 mM (non-inducing) MgCl_2_ (Thaipisuttikul et al., 2014). D-amino acids were added as required to support the growth of auxotrophic *P. aeruginosa* strains, as described above.

Antibiotics were used at the following concentrations: for *P. aeruginosa*, 15 µg/mL of chloramphenicol (Cm), 2,000 µg/mL of streptomycin (Sm), and 30 µg/mL of gentamicin (Gm). For *E. coli*, 15 µg/mL Gm and 50 µg/mL Sm were used. All antibiotics were purchased from Sigma-Aldrich, Inc.

### 2.2 DNA manipulation and construction of mutants

Genomic DNA from *P. aeruginosa* was extracted from 1 mL of stationary-phase culture using the Wizard^®^ Genomic DNA Purification Kit (Promega), following the manufacturer’s instructions. Plasmid DNA from *E. coli* was extracted using the GeneJET Plasmid Miniprep Kit (Thermo-Fisher Scientific), according to the manufacturer’s guidelines. PCR reactions were carried out using either GoTaq^®^ DNA polymerase (Promega) or PrimeSTAR^®^ HS DNA polymerase (Takara), selected based on fidelity requirements, and performed under the conditions recommended by the respective manufacturers. **Supplementary Table 1** lists the primers used in this study, which were designed based on the *P. aeruginosa* PAO1 genome (Accession No. NC_002516). FastDigest^®^ restriction enzymes, FastAP™ Alkaline Phosphatase (Thermo-Fisher Scientific) and T4 DNA ligase (Promega) were used as recommended by the supplier.

Gene deletions were performed by allelic exchange using the suicide plasmids pEX18Gm (Hoang et al., 1998) and pKNG101 (Kaniga et al., 1991) (**Table 1**). Upstream and downstream regions (~1 kb each) flanking the target gene were PCR-amplified from PAO1 genomic DNA using primers described in **Supplementary Table 1** which contained restriction sites for cloning. PCR products were digested with restriction enzymes and ligated into linearized pEX18Gm or pKNG101 vectors. The resulting ligation product was used to transform *E. coli* SM10 λ*pir* via electroporation. Transformants were selected on LB agar with 15 μg/mL Gm (for pEX18Gm) or 50 μg/mL Sm (for pKNG101). Colony PCR using universal primers targeting the vector (**Supplementary Table 1**) were used to screen the colonies containing the plasmid with the correct insertion of fragments. Plasmid DNA from positive clones was purified and verified by sequencing, yielding plasmids pEX18Gm(UP-Δ*pagP*-DN), pKNG101(UP-Δ*htrB1*-DN), pKNG101(UP-Δ*htrB2*-DN), pKNG101(UP-Δ*pagL*-DN) (**Table 1**).

Recombinant plasmids were introduced into *P. aeruginosa* by electroporation (derivatives of pEX18Gm) or conjugation (derivatives of pKNG101) and chromosomal integration occurred via homologous recombination. For pKNG101, merodiploids were selected on LB agar containing 10 mM D-glutamate, 10 mM D-alanine, 25 μg/mL Cm, and 2,000 μg/mL Sm at 37 °C for 48 h; for pEX18Gm, 50 μg/mL Gm were used. First crossover events were confirmed by PCR. Merodiploid were grown in LB with 15% sucrose, 10 mM D-glutamate, and 10 mM D-alanine for 6 h at 37 °C with shaking and then plated to select the second crossover. Colonies sensitive to Cm and Sm (pKNG101) or Gm (pEX18Gm) were screened by PCR and confirmed by sequencing to verify deletion of the wild-type allele.

### 2.3 RNA extraction and RT-qPCR reactions

To evaluate the expression of *P. aeruginosa* genes *htrB1*, *htrB2*, *pagP* and *pagL*, two-step reverse transcription quantitative PCR (RT-qPCR) were performed using TaqMan probes from the Universal Probe Library^®^ (UPL; Roche, Germany) with gene-specific primers (**Supplementary Table 2**). Primers were designed using the Roche Universal Probe^®^ service or the University of the Sunshine Coast’s primer design tool (https://primers.neoformit.com/). Total RNA was extracted from logarithmic-phase cultures using the High Pure RNA Isolation Kit (Roche), and diluted to 50 ng/μL. For cDNA synthesis, 200 ng of RNA was reverse-transcribed using random hexamers (60 μM) and the Transcriptor First Strand cDNA Synthesis Kit (Roche). Subsequent qPCR was performed using 2 μL cDNA, gene-specific primers and UPL probes with the LightCycler^®^ 480 Probes Master Kit (Roche). Reactions were run on a LightCycler^®^ 480 Instrument II under the following conditions: 95 °C for 10 min (4.4 °C/s), 45 cycles of 95 °C for 10 s (4.4 °C/s), 60 °C for 45 s (2.2 °C/s), 72 °C for 1 s (4.4 °C/s), and a final cooling at 40 °C for 30 s (1.5 °C/s). Relative expression was calculated using the Livak method (Livak and Schmittgen, 2001) and normalized to the reference gene *rpoS*. All RNA extractions and RT-qPCR experiments were performed in duplicate across three independent biological replicates.

### 2.4 Osmolysis assays in water

To evaluate the persistence of *P. aeruginosa* PAO1 and its auxotrophic variants in the general environment, survival assays were conducted in sterile distilled water. Strains were grown in LB supplemented with the required D-amino acids for 16 h, harvested by centrifugation (5,000 × g, 10 min, 4 °C), washed and resuspended in water to an OD_600_ of 0.5. Bacterial suspensions were incubated at 26 °C with shaking (180 rpm) and samples were collected on multiple days to determine viability. All experiments were performed in triplicate.

### 2.5 LPS isolation and lipid A MALDI-TOF MS analysis

LPS was extracted from *P. aeruginosa* cultures in exponential phase using the Tri-Reagent^®^ method (Yi and Hackett, 2000). Bacteria were harvested by centrifugation (4,000 rpm, 10 min), washed with saline solution, and lyophilized overnight using a Labogene Scanvac CoolSafe Touch Freeze Dryer. Then, 30 mg of lyophilized bacteria were homogenized in 200 μL of TRIZol™ (ThermoFisher, USA) followed by phase separation with 600 μL of chloroform and centrifugation (12,000 × g, 10 min). The aqueous phases from three extractions were pooled, frozen at –80 °C and lyophilized. The resulting powder was resuspended in 1.5 mL of 0.375 M MgCl_2_ in 95% ethanol at –20 °C. After centrifugation (12,000 × g, 15 min), the white sediment was resuspended in 600 μL of Milli-Q water. LPS concentration was determined by quantifying Kdo using a modified Lam et al. method (Lam et al., 2014). Briefly, 250 μL of purified LPS was hydrolyzed with 0.5 N H_2_SO_4_ at 100 °C for 30 min, cooled, and centrifuged. The supernatant was sequentially treated with NaIO_4_, NaAsO_2_, and thiobarbituric acid, and absorbance at 548 nm was measured after DMSO addition in a conventional ELISA plate reader. LPS was assessed using 16% sodium dodecyl sulfate-polyacrylamide gel electrophoresis (SDS-PAGE) with silver staining, as previously described (Hitchcock and Brown, 1983).

Lipid A of *P. aeruginosa* PAO1 ΔΔΔ and their mutant derivatives, grown as described above, was extracted using the MBT Lipid Xtract Kit™ (Bruker, Germany) following the manufacturer’s recommendations. Briefly, a 1 μL inoculation loop of bacterial biomass was suspended in 50 μL of MBT Lipid Xtract Hydrolysis Buffer, and 4 μL of the suspension was heat-treated at 90 °C for 10 min to induce mild acid hydrolysis and release of LPS. The resulting pellet was washed and the dried pellet was then resuspended in 5 μL of matrix solution for matrix-assisted laser desorption/ionization time-of-flight mass spectrometry (MALDI-TOF MS) analysis. Lipid A mass spectra were acquired using a MALDI Biotyper^®^ Sirius System (Bruker Daltonics, Inc.) in negative linear mode (500-3,000 *m/z*). Mass peaks were recorded in 100-shot steps (total 400 shots). Calibration with a lipid A standard was performed daily in all assays. Mass errors were not higher than ± 300 ppm.

### 2.6 TLR4 stimulation assays

HEK293-Blue™ cells expressing murine TLR4 (mTLR4) containing a reporter gene for Secreted Embryonic Alkaline Phosphatase (SEAP) (Invivogen, France) were cultured in DMEM with 5% of fetal calf serum in 25 cm^2^ sterile flasks. Cells were maintained at 37 °C with 5% CO_2_, and subcultured every 3–4 days upon reaching 70-80% confluence. Cells between passages 4 and 25 were used for LPS stimulation assays. When required, they were seeded into 96-well plates and incubated for 24 h before stimulation with serial dilutions of purified LPS (10 ng/mL) or heat-killed bacterial cell preparations adjusted to an OD_600_ of 0.1 in PBS. After 24 h of stimulation, SEAP activity in supernatants was measured using QUANTI-Blue™ reagent (InvivoGen, France) following the manufacturer’s instructions. The reaction was developed for 10–40 min, and the absorbance was read at 630 nm in a conventional ELISA reader.

### 2.7 Animal experiments

All animal procedures adhered to European Union Directive 2010/63/EU and Spanish Royal Decree 53/2013, and were approved by the Animal Experimentation Ethics Committee of Complexo Hospitalario Universitario de A Coruña (CHUAC) and Axencia Galega de Calidade Alimentaria (AGACAL) of Xunta de Galicia, under permit number 15002/2018/007. A total of 96 BALB/c mice (56 males and 40 females, 8–12 weeks old) were bred and housed under specific pathogen-free conditions at the CTF-XXIAC (SERGAS), with access to pelleted chow and water *ad libitum*.

For mouse inoculations, bacteria were cultured in the appropriate medium at 37 °C with shaking for 16 h, diluted 1:50 in fresh medium and grown to an OD_600_ of 0.7. Cells were harvested (5,000 × g, 15 min, 4 °C), washed and resuspended in 0.85% saline solution to the desired concentration for *in vivo* use. An aliquot of this suspension was plated to determine the number of viable bacteria. To assess virulence attenuation, mice were randomly divided into groups and inoculated either intraperitoneally (IP; seven groups of eight mice per group) or IN (seven groups of six mice per group) with a high dose (about 10^9^ CFU) of the vaccine candidates and monitored daily for survival over a 7-day observation period. IP administration was performed by injecting 100 µL of bacterial suspension into mice, previously anesthetized by sevoflurane inhalation, whereas IN administration involving inoculating 10 µL of bacterial suspension evenly distributed between both nostrils. To evaluate vaccine immunogenicity, thirty-six mice were randomly divided into five groups (seven mice per vaccine group and eight mice in the saline control group). Each group received two IN doses of the vaccine candidates administered 14 days apart, while the control group received saline under the same conditions. Blood samples were collected via submandibular vein puncture, allowed to clot, centrifuged, and the sera were stored at –80 °C until IgG titers were determined. Vaginal fluid lavage (VFL) samples were obtained by flushing the vagina of female mice twice with 50 μL of sterile saline containing a protease inhibitor cocktail (Sigma-Aldrich) and stored at –80 °C for IgA titer quantification. For the acute lung infection model, four weeks after the last immunization dose, mice (eight mice per group) were anesthetized via sevoflurane inhalation and IN-inoculated with 1 × 10^6^ CFU of *P. aeruginosa* PA14. To prevent the expulsion of inoculum and facilitate its migration to the alveoli, mice remained in an inclined position under anesthesia for an additional 2 min. Mice were monitored daily for 7 days with individualized follow-ups, and clinical symptoms (*e.g.*, inactivity, lack of response, weight loss, dehydration, piloerection, labored breathing, nasal discharge, skin lesions) were assessed. Each symptom was scored as follows: 0 (normal), 1 (slight deviation), 2 (moderate deviation) or 3 (severe deviation). Humane endpoints (total score ≥ 14), irrecoverable weight loss (> 25%), or severe clinical signs (*e.g.*, respiratory or neurological distress) prompted euthanasia by IP overdose of thiopental (Morton and Griffiths, 1985; Hawkins et al., 2011). The immunization-challenge experiment, including the immunization schedule, booster, and observation period, lasted a total of 7 weeks from the first immunization to the conclusion of the study.

### 2.8 Indirect ELISA

For indirect ELISA, 96-well plates (Corning, USA) were coated with live or formalin-inactivated *P. aeruginosa* strains in 100 mM carbonate-bicarbonate buffer, pH 9.6, at 4 °C for 16 h. After blocking with 5% skim milk (Sigma-Aldrich), serial dilutions of mouse serum or VFL were added and incubated at 4 °C, in accordance with the previously described protocol (Fuentes-Valverde et al., 2022). The wells were then incubated with HRP-conjugated anti-mouse IgG (Sigma-Aldrich) or IgA (Bethyl Laboratories) in DMEM supplemented with 10% fetal calf serum at 37 °C for 2 h. After washing, the reaction was developed with 3,3’,5,5’-tetrametilbenzidina (TMB, ThermoFisher Scientific). Absorbance at 450 nm was measured using a NanoQuant Infinite^®^ 200 Pro plate reader (Tecan). The endpoint titer was defined as the highest dilution with an absorbance at least 0.1 above the blank.

### 2.9 Statistical analysis and software

GraphPad Prism (v6.01) was used for graph creation and statistical analysis. Means were compared using Student’s *t* test with Welch’s correction, multiple *t*-tests with Holm-Sidak adjustment, or ANOVA followed by Dunnett’s *post-hoc* test. The Mann-Whitney *U* test was used for two-group comparisons, and the Kruskal-Wallis test for comparisons involving three or more groups. Survival analysis was performed using the Mantel-Cox log-rank test, with significance levels adjusted by the Holm-Sidak method. Genetic sequence analysis was conducted using Vector NTI, MALDI-TOF MS spectra were processed with Clover MS Data Analysis Software (Clover Bioanalytical Software, Granada, Spain), and lipid A structures variants were drawn using ChemSketch.

## 3 Results

### 3.1 Construction and phenotypic characterization of novel LPS mutants

Lipid A of the PAO1 ΔΔΔ was modified by single inactivation of the genes encoding lipid A-biosynthesis enzymes (*htrB1* and *htrB2*) and lipid A-modifying enzymes (*pagP* and *pagL*). Additionally, double mutants were generated by consecutive inactivation of *htrB1* or *htrB2* and *pagP.* The resultant mutants were sucrose-resistant and antibiotic-sensitive, and gene deletions were confirmed by PCR (**Supplementary Figure 2**) and sequencing (data not shown). These mutants are expected to alter the number of acyl chains in the lipid A molecule (**Supplementary Figure 1B**).

**Figure 1:**
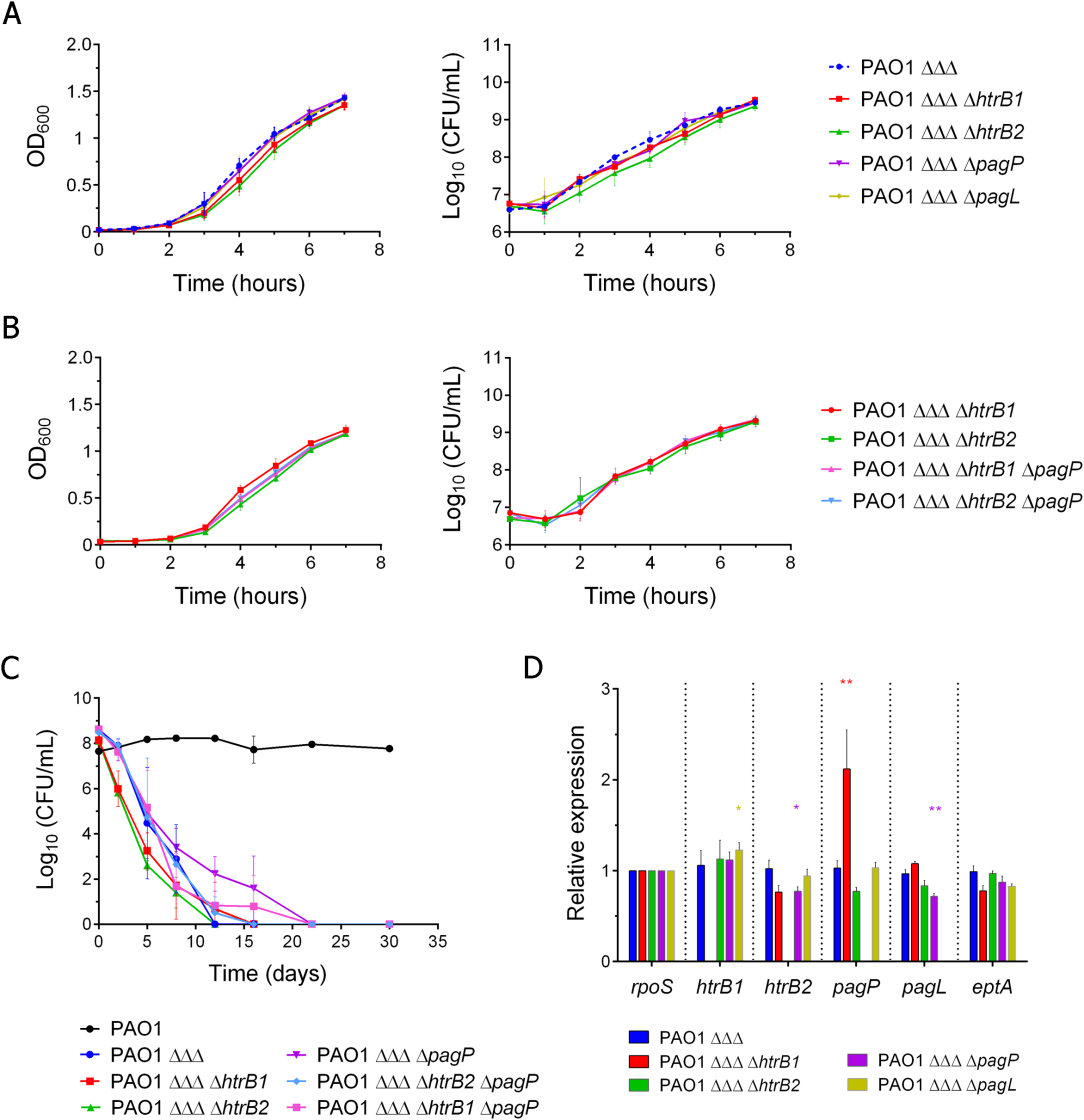
Characterization of the PAO1 ΔΔΔ strain and LPS-modified mutants. Growth curves and viability of the PAO1 ΔΔΔ strain and LPS mutants with single deletions **(A)** or double deletions **(B)**, cultured in LB medium supplemented with D-glutamate and D-alanine. Data are presented as the mean ± SD from three independent experiments. **(C)** Persistence of LPS-modified PAO1 ΔΔΔ derivatives in the environment. Viable cell counts (Log_10_ CFU/mL) for the wild-type PAO1 strain and LPS mutants derived from the double auxotroph PAO1 ΔΔΔ maintained in distilled water at 26 °C with agitation over 30 days. Data represent the mean ± SD of three independent experiments. **(D)** Relative expression levels of genes involved in lipid A modification (*htrB1*, *htrB2*, *pagP*, *pagL* and *eptA*) analyzed by RT-qPCR and compared to the PAO1 ΔΔΔ strain. Logarithmic-phase cultures grown in LB medium supplemented with D-glutamate and D-alanine were used for total RNA extraction. Expression was quantified using the Livak method (2^−ΔΔCt^). *p < 0.05, **p < 0.01, one-way ANOVA, with Dunnet’s *post-hoc* test.

The growth rate and bacterial survival of the parent strain and mutant derivatives were evaluated in LB medium supplemented with D-glutamate and D-alanine. No significant differences were observed in the single or double mutants compared to the parental strain (**Figures 1A, B**). However, PAO1 Δ*htrB1* and PAO1 Δ*htrB2* exhibited a slight, non-significant delay at the beginning of the exponential growth phase (**Figure 1A**), which had no apparent effect on the survival.

Environmental safety was assessed via osmotic stability assays in water. Strain PAO1 remained viable for at least 30 days. However, PAO1 ΔΔΔ and the corresponding mutant derivatives exhibited a progressive loss of viability in water within the first 25 days (**Figure 1C**), evidencing a reduced persistence compared to the wild-type PAO1 strain.

Mutation of enzymes that participate in the lipid A biosynthesis or modification may alter the expression of other related genes. Thus, we decided to evaluate the expression of the targeted genes in the wild-type and mutant derivatives using RT-qPCR. As expected, no expression of the targeted genes was detected in the corresponding deletion mutants (**Figure 1D**), confirming the correct preparation of the strains. However, the expression levels of several genes varied across the different mutant strains (**Figure 1D**). *pagP* was significantly overexpressed (approximately a 2.12-fold increase) when *htrB1* was inactivated. In contrast, the expression of *pagP* and *pagL* was reduced when *htrB2* was knocked out. Moreover, the expression of *htrB2* and *pagL* was significantly reduced when *pagP* was inactivated, while *htrB1* expression was significantly increased upon *pagL* inactivation. No significant differences in *eptA* expression were observed between the mutants and the parent strain.

### 3.2 Structural analysis of genetically modified LPS

**Figure 2:**
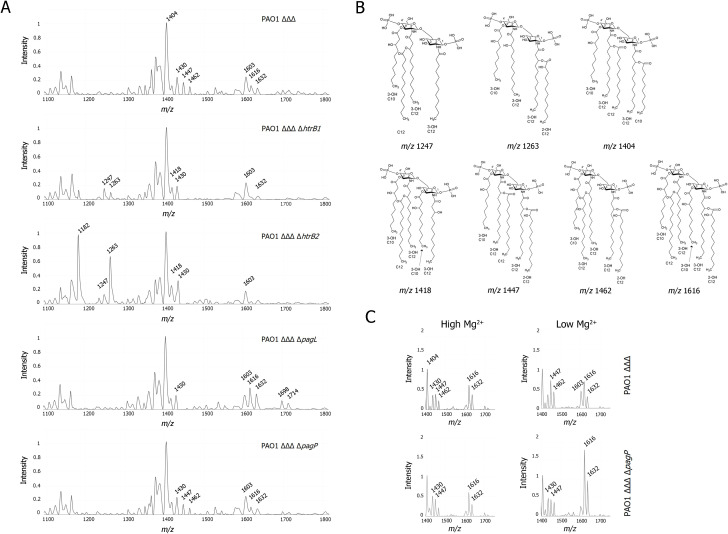
Structural analysis of lipid A in the PAO1 ΔΔΔ vaccine strain and its acylase- and deacylase-deficient derivatives. **(A)** MALDI-TOF MS spectra in negative-ion mode for the following strains: PAO1 ΔΔΔ, PAO1 ΔΔΔ Δ*htrB1*, PAO1 ΔΔΔ Δ*htrB2*, PAO1 ΔΔΔ Δ*pagL*, PAO1 ΔΔΔ Δ*pagP*. The displayed *m/z* range (1100-1800) shows the main lipid A ion species. Intensity is expressed as relative intensity normalized to the highest intensity peak in the spectra (peak *m/z* 1404). **(B)** Presumed structures for lipid A variants of the spectra peaks identified in PAO1 ΔΔΔ mutants and their LPS derivatives: tetra-acylated (*m/z* 1247, 1263), penta-acylated (*m/z* 1404, 1418, 1447, 1462) and a hexa-acylated species (*m/z* 1616). Structures were drawn using ChemSketch software. **(C)** Lipid A structural analysis of the PAO1 ΔΔΔ and PAO1 ΔΔΔ Δ*pagP* strains cultivated in minimal N medium under inducing (low Mg^2+^, 8 μM MgCl_2_) and non-inducing (high Mg^2+^, 1 mM MgCl_2_) conditions. MALDI-TOF MS spectra in negative ion mode. The *m/z* 1616 and 1632 peaks correspond to non-palmitoylated hexa-acylated lipid A variants.

LPS was extracted from PAO1 ΔΔΔ strain and mutant derivatives using the Tri-reagent method (Yi and Hackett, 2000). Samples were analyzed on SDS-PAGE gels, confirming successful LPS extraction and the absence of protein contamination (data not shown), and further analyzed by MALDI-TOF MS in negative-ion mode (**Figure 2A**). Results and proposed structures are shown in **Figure 2B** and extended in **Supplementary Figure 3**. It is important to note that structural assignments based solely on intact *m/z* values obtained from single-stage MALDI-TOF MS are inherently tentative and should be considered putative (Park et al., 2017). The lipid A of PAO1 ΔΔΔ strain displayed high heterogeneity, with a dominant peak at *m/z* 1404. In agreement with assignments previously reported for stationary-phase *P. aeruginosa* PAO1 (Adamiak et al., 2021), this peak was tentatively attributed to an atypical penta-acylated, bis-phosphorylated lipid A species containing a shorter 10-carbon acyl chain (C10) as the secondary acylation at position 2 (Hernández-García et al., 2024; Moscoso et al., 2025). However, this assignment remains uncertain, as endogenous cardiolipin (specifically the CL 68:2 isoform) could generate an identical [M-H]^−^ ion at *m/z* 1404.2 and therefore cannot be excluded (Yang et al., 2020). Additional minor peaks at *m/z* 1430, 1447, and 1462 presumably correspond to canonical penta-acylated variants with a 12-carbon acyl chain (C12) or a hydroxylated C12 (2-OH C12) as the secondary acyl chain at position 2, consistent with established structural studies (Ernst et al., 2003; Hittle et al., 2015; Yang et al., 2020). The *m/z* 1462 species contains an additional hydroxyl group (*m/z* + 16) on the laurate at position 2’, introduced by the LpxO1 enzyme (Thaipisuttikul et al., 2014; Yang et al., 2020; Hofstaedter et al., 2024a). A peak at *m/z* 1418 indicated a penta-acylated variant with two 3OH-C10 and two 3OH-C12 primary fatty acids and a C12 secondary acyl chain. Finally, hexa-acylated forms at *m/z* 1603, 1616 and 1632 likely resulted from additional acylation (C12, 2OH-C12, 3OH-C10) of the penta-acylated variants, consistent with previously characterized lipid A profiles (Hittle et al., 2015; Park et al., 2017; Hernández-García et al., 2024). Such variability was recently reported for PAO1 Δ*murI* (Moscoso et al., 2025).

Inactivation of *htrB1* depleted peaks at *m/z* 1447, 1462, and 1616, corresponding to penta- and hexa-acylated lipid A variants, and new peaks appeared at *m/z* 1247 and 1263 (**Figure 2**). The *m/z* 1247 ion likely reflects a tetra-acylated form derived from the *m/z* 1462 following loss of a 2OH-C12 chain (Δ*m/z* 216) (Park et al., 2017). Similarly, inactivation of Δ*htrB2* displayed an abundant peak at *m/z* 1263, likely resulting from the loss of a C12 acyl chain (Δ*m/z* 184) from the *m/z* 1447 variant (Hittle et al., 2015). Notably, this mutant also exhibited a major ion at *m/z* 1182, which could be associated with a mono-phosphorylated tetra-acylated variant. In the strain PAO1 ΔΔΔ Δ*pagL*, peaks at *m/z* 1447 and 1462 were absent. Additionally, a slight increase in the relative intensity of the *m/z* 1616 and 1632 peaks was observed (**Figure 2**).

**Figure 3:**
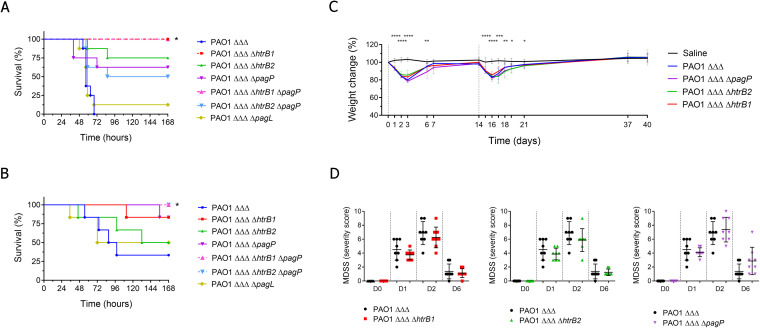
*In vivo* virulence attenuation of PAO1 ΔΔΔ derivatives with modified LPS. Survival curves of BALB/c mice inoculated with 2 × 10^9^ CFU of PAO1 ΔΔΔ or its LPS-modified derivatives via intraperitoneal (*n* = 8 per group) **(A)** or intranasal (*n* = 6 per group) **(B)** routes. *p < 0.05, Log-rank (Mantel-Cox) test, compared to PAO1 ΔΔΔ. **(C)** Effect of IN immunization on body weight changes. The graph shows the average weight variation expressed as a percentage. Dashed lines indicate vaccination days (0 - experiment start, 14). *p < 0.05, **p < 0.01, ***p < 0.001, ****p < 0.0001, *t*-test using the Holm-Sidak method, with α = 5%. **(D)** Assessment of murine disease severity score (MDSS) after administration of different vaccine candidates.

Initial analysis showed similar spectra between the parental strain and the Δ*pagP* mutant, as well as between the acyltransferases-deficient mutants (Δ*htrB1* and Δ*htrB2*) and their respective *pagP*-inactivated counterparts (**Figure 2A**; **Supplemtary Figure 3**). The only notable difference was a slightly reduction in the peaks at *m/z* 1182 and 1263 in the PAO1 ΔΔΔ Δ*htrB2* Δ*pagP* double mutant compared with the PAO1 ΔΔΔ Δ*htrB2* mutant. Since *pagP* expression is regulated by the PhoPQ two-component system (TCS), experiments under inducing (low Mg²^+^) and non-inducing (high Mg²^+^) conditions were performed. Under high Mg²^+^ conditions, spectra of the parental PAO1 ΔΔΔ strain and the Δ*pagP* mutant were similar. Under Mg²^+^ limitation (inducing conditions), the PAO1 ΔΔΔ Δ*pagP* mutant showed an increase of the relative abundance of *m/z* 1616 and 1632 peaks, consistent with non-palmitoylated hexa-acylated forms and PhoPQ-dependent regulation (**Figure 2C**). The *m/z* 1632 ion is interpreted as a hexa-acylated variant with a +16 *m/z* shift, suggesting an additional hydroxylation of the laurate group at position 2’, potentially mediated by the LpxO1 enzyme (Hofstaedter et al., 2024a).

### 3.3 *In vivo* assessment of strain attenuation

To assess the impact of LPS modifications on strain attenuation, *in vivo* studies were conducted in a murine model to assess virulence attenuation and safety: fifty-six female BALB/c mice were randomly divided into seven groups (*n* = 8 per group) and inoculated IP with a high dose on the order of 10^9^ CFU of the PAO1 ΔΔΔ strain or its derivatives. Mice were monitored daily for survival over a 7-day period. The PAO1 ΔΔΔ and the PAO1 ΔΔΔ Δ*pagL* strains were highly virulent, causing 100% and 87.5% mortality (7 out of 8 mice) within 70 h, respectively. In contrast, reduced mortality was observed in mice receiving the mutants with *htrB2* or *pagP* deletions: 50% for Δ*htrB2* Δ*pagP* (4 out of 8 mice), 37.5% for Δ*pagP* (3 out of 8 mice), and 25% for Δ*htrB2* (2 out of 8 mice). Notably, all mice inoculated with the PAO1 ΔΔΔ Δ*htrB1* and PAO1 ΔΔΔ Δ*htrB1* Δ*pagP* strains survived and showed no signs of disease (**Figure 3A**), indicating attenuation of virulence in these strains. Due to limited attenuation of PAO1 ΔΔΔ Δ*pagL* in mouse infection models, this mutant was excluded from further analysis. This observation may be consistent with a previous report showing that loss of PagL-mediated lipid A deacylation enhances the host cytokine response (Hofstaedter et al., 2024b).

Since IN administration is an attractive route for vaccination, we further assessed *in vivo* safety by this route. Therefore, forty-two male BALB/c mice were randomly divided into seven groups (*n* = 6 per group) and IN inoculated with a high dose (~10^9^ CFU) of PAO1 ΔΔΔ or its mutant derivatives, after which survival was monitored daily for 7 days. The PAO1 ΔΔΔ strain caused 67% mortality (4 out of 6 mice) within 55–96 h. In contrast, survival improved in mice receiving the mutants: 50% for Δ*htrB2* and Δ*pagL*, 83.3% for Δ*htrB1* and Δ*pagP*, and 100% for the double mutants (Δ*htrB1* Δ*pagP* and Δ*htrB2* Δ*pagP*) (**Figure 3B**), suggesting enhanced attenuation in the latter strains. Next, the impact of IN administration on the body weight, appearance, or behavior of the mice was evaluated. Female BALB/c mice (*n* = 8 per group) were IN vaccinated with approximately 3.5 × 10^8^ CFU of each strain using a two-dose schedule with a 14-day interval (**Supplementary Table 3**). After the first dose, transient weight loss (up to 20%) was observed, along with temporary signs such as piloerection, reduced grooming, and mild hypoactivity. However, all mice recovered within 7 days. No significant differences were observed between the native PAO1 ΔΔΔ and its LPS-mutant derivatives regarding weight or behavior (**Figures 3C, D**).

### 3.4 Bioactivity of novel LPS mutants

**Figure 4:**
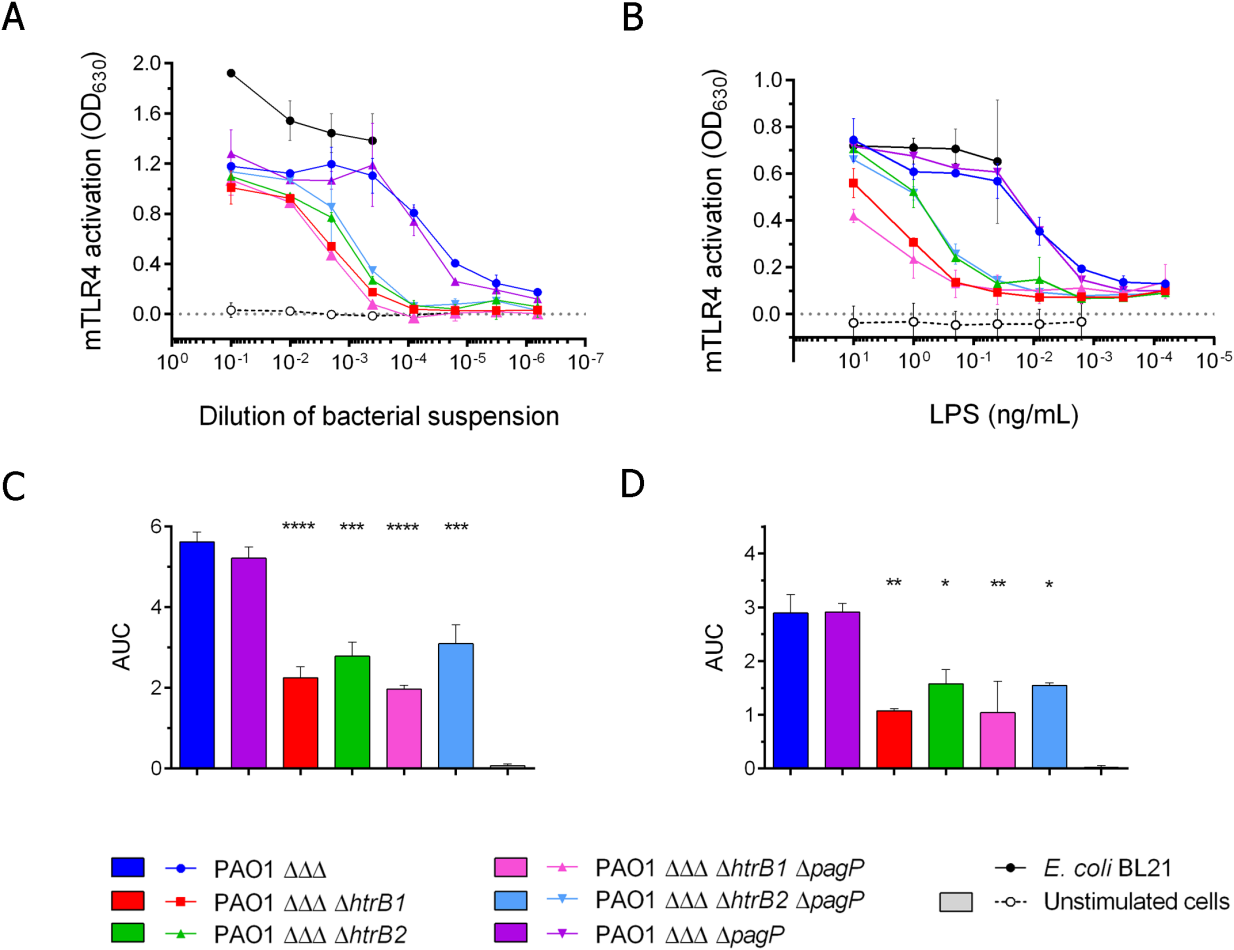
TLR4 activation by whole cell preparations **(A, C)** and purified LPS **(B, D)** from PAO1 ΔΔΔ and its genetically modified LPS derivatives. HEK293-Blue cells expressing mTLR4 were stimulated with serial dilutions of **(A)** heat-killed bacterial suspensions (OD_600_ = 0.1) or **(B)** purified LPS (10 ng/mL) for 24 h at 37 °C, 5% CO_2_. Alkaline phosphatase activity was measured at OD_630_ in the culture supernatants. The corresponding area under the curve (AUC) values are shown in **(C, D)**, respectively. Data are presented as mean ± SD from two independent experiments. *p < 0.05, **p < 0.01, ***p < 0.001, ****p < 0.0001 compared to PAO1 ΔΔΔ (One-way ANOVA with Dunnet’s *post-hoc* test).

To understand whether the lipid A modifications affect lipid A activity in our vaccine strain, whole bacteria cells (**Figure 4A**) or purified LPS preparations (**Figure 4B**) from the mutants and the parental strain were used to stimulate HEK293-Blue cells expressing mTLR4. All mutant derivatives, except PAO1 ΔΔΔ Δ*pagP*, exhibited an approximately 100-fold reduction in mTLR4 activation compared to the parental strain, indicating diminished LPS bioactivity in these mutants. Area under the curve (AUC) analysis from two independent experiments confirmed significantly lower activity in all deletion mutants compared to the parent strain, except PAO1 ΔΔΔ Δ*pagP* (**Figures 4C, D**).

### 3.5 Development of immune response and protection

**Figure 5:**
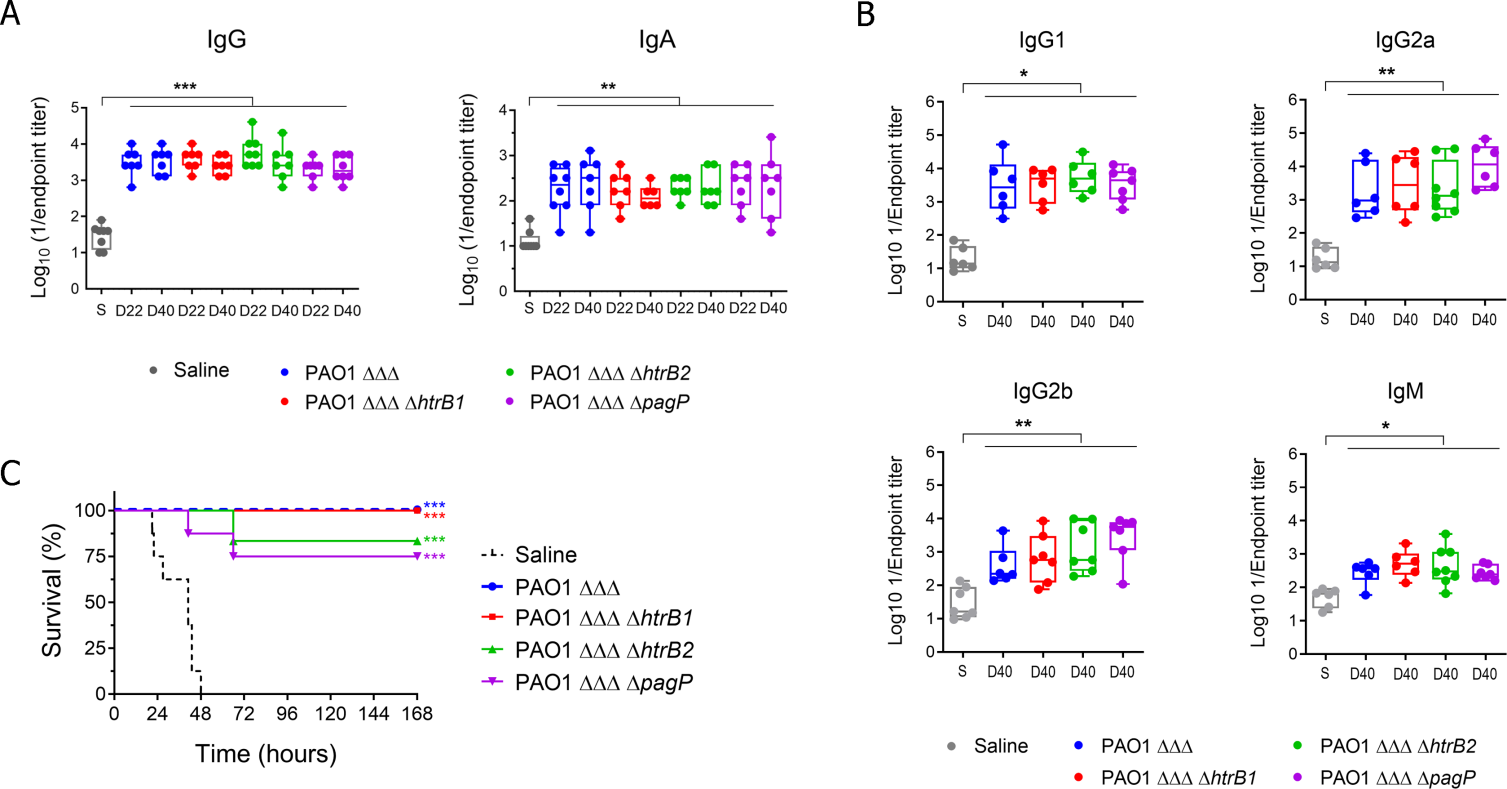
**(A, B)** Humoral response after IN immunization with PAO1 ΔΔΔ derivatives containing modified LPS. Serum IgG titers and vaginal lavage IgA (*n* = 7 per vaccine group; *n* = 8 for saline control group), as well as serum IgG subclasses and IgM (*n* = 6 per vaccine group; *n* = 7 for saline control group) were measured in BALB/c mice immunized with PAO1 ΔΔΔ or its modified LPS derivatives. *p < 0.05, **p < 0.01, ***p < 0.001, Kruskal-Wallis test, compared to the saline group. S, saline; D, day. **(C)** Protection against acute pneumonia caused by *P. aeruginosa*. Survival curves of BALB/c mice (*n* = 8 per group) IN immunized with PAO1 ΔΔΔ and its modified LPS derivatives, followed by IN challenge with PA14 (1 × 10^6^ CFU). ***p < 0.001, Log-rank test (Mantel-Cox) test, compared to the saline control group.

To evaluate the induction of systemic and mucosal antibody responses, mice were IN-immunized, and serum and VFL samples were collected on days 22 and 40, corresponding to one and four weeks after the second immunization, respectively. ELISA analysis revealed that vaccinated mice showed significantly increased serum IgG levels (2 log units; *p* < 0.001) and mucosal IgA levels in VFL (over 1 log unit; *p* < 0.01) compared to the saline control group (**Figure 5A**). No significant differences were observed among the vaccine groups or between the two time points (days 22 and 40) within the same group. Furthermore, all vaccine candidates induced high levels of IgG subclasses (IgG1, IgG2a, and IgG2b) and IgM after the second immunization compared to the saline control group, with no significant differences detected among vaccine groups (**Figure 5B**).

Finally, protective efficacy was evaluated using a murine model of acute lung infection. While PAO1 is a well-characterized laboratory strain commonly used in vaccine studies, PA14 is a more virulent clinical isolate with a distinct repertoire of toxins and virulence factors (Lee et al., 2006); therefore, PA14 was included to assess vaccine efficacy against a heterologous strain. Four weeks after the final immunization, mice were IN challenged with the hypervirulent PA14 strain (~10^6^ CFU), and survival was monitored for one week. All non-vaccinated mice succumbed to the infection within 48 h. Among vaccinated mice, survival rates were 75% for Δ*pagP*, 87.5% for Δ*htrB2*, and 100% for PAO1 ΔΔΔ and Δ*htrB1*. The log-rank test revealed no significant differences between vaccination groups, suggesting that the LPS-modified strains preserve the protective efficacy of the vaccine strain (**Figure 5C**).

## 4 Discussion

*P. aeruginosa* is classified as a high-priority pathogen by the WHO (Jesudason, 2024), and was ranked as the sixth leading cause of deaths associated with antibiotic resistance in 2019 (Ikuta, 2022). The continuous rise in antimicrobial resistance represents a critical global health concern, emphasizing the urgent need for new therapeutic strategies (Tacconelli et al., 2018). In this context, vaccination offers a promising approach, preventing infections that would otherwise require antibiotic treatment and thereby reducing the selective pressure that drives resistance development (Lopez-Siles et al., 2021). Despite numerous efforts, no licensed vaccine is currently available for human use against *P. aeruginosa* infections. Live-attenuated vaccines, particularly those based on auxotrophic strains with targeted gene inactivation in pathways essential for cell wall biosynthesis, have demonstrated robust immunogenicity and protective efficacy (Cabral et al., 2017, 2020; Fuentes-Valverde et al., 2022). However, in our study, IN immunization with high doses of the vaccine candidate led to adverse effects associated with excessive inflammation. To mitigate this reactogenicity and improve vaccine safety, we focused on targeting enzymes responsible for lipid A biosynthesis and modification, aiming to reduce the inflammatory properties of LPS.

The design of new *P. aeruginosa* vaccine candidates involved the inactivation of genes that modulate lipid A structure, including *htrB1* and *htrB2*, which encode acyltransferases, as well as *pagL* (encoding PagL deacylase) and *pagP* (encoding palmitoyl transferase). Structural analysis of the lipid A from PAO1 ΔΔΔ derivatives revealed different profiles according to the enzyme inactivation. Native LPS spectra showed significant heterogeneity, including both presumed penta-acylated and hexa-acylated variants. In *P. aeruginosa*, lipid A structure differs across isolates: environmental and laboratory-adapted strains typically produce penta-acylated lipid A, whereas pwCF isolates often generate hexa-acylated forms via PagP, enhancing human TLR4-driven inflammation (Ernst et al., 1999; Thaipisuttikul et al., 2014; Hofstaedter et al., 2024a). In contrast, PagL-deficient pwCF isolates generate hepta-acylated lipid A with reduced inflammatory activity compared with PAO1 (Cigana et al., 2009; Hofstaedter et al., 2024b).

Structural analysis of lipid A from the PAO1 ΔΔΔ Δ*pagL* mutant revealed the absence of the predominant presumed penta-acylated variants, as well as hepta-acylated lipid A species that would be expected if PagP were functionally active in this mutant. In contrast, lipid A profiles from the vaccine candidate PAO1 ΔΔΔ Δ*pagP* under inducing conditions (low Mg^2+^) closely resembled those reported by Thaipisuttikul et al (Thaipisuttikul et al., 2014), showing increased signals at *m/z* 1616 and 1632 (non-palmitoylated forms), compared to non-inducing conditions (high Mg^2+^). However, the peaks associated with hexa-acylated lipid A species generated by PagP and PagL activity, were scarcely detected in PAO1 ΔΔΔ background. This indicates that, although *pagP* expression was detected, PagP-mediated acylation was either a minor event in our strains under the experimental growth conditions used or occurred at levels below the detection threshold of the method. We also acknowledge the inherent limitations of MALDI-TOF MS for the detection and resolution of higher *m/z* lipid A species. Complementary approaches, such as LC-MS/MS or GC-MS/MS, would provide enhanced sensitivity and improved structural resolution for species with *m/z* values above 1800. Nevertheless, no significant differences in TLR4/MD-2 activation were observed, likely due to the dominant species at *m/z* 1404, which appears to exert a comparable immunostimulatory effect. Accordingly, inactivation of *pagP* resulted in a bioactivity profile similar to that of the parent strain. These findings are consistent with previous observations in *Bordetella bronchiseptica*, where inactivation of *pagP*, which resulted in the loss of the secondary palmitate chain at the 3’ position of lipid A (Preston et al., 2003), had no noticeable effect on TLR4 activation (Perez-Ortega et al., 2021). The effect of Mg²^+^ on *pagL* expression in *P. aeruginosa* remains unresolved: Geurtsen et al. (2005) reported no expression changes under varying Mg²^+^ levels (Geurtsen et al., 2005), whereas Ernst et al. (2006) observed *pagL* induction at low Mg²^+^ concentrations (Ernst et al., 2006). In the PAO1 ΔΔΔ Δ*pagP* mutant, no increase in PagL activity was detected under low Mg²^+^ conditions, as indicated by the accumulation of *m/z* 1616 peak and the absence of an increased *m/z* 1447 peak, which would be consistent with deacylase activity (Ernst et al., 2003).

HtrB1 and HtrB2 incorporate secondary acyl chains into lipid A at positions 2 (2OH-C12) and 2’ (C12), respectively (Moskowitz and Ernst, 2010; Hittle et al., 2015). Although non-essential for *P. aeruginosa* viability, deletion of *htrB1* and *htrB2* reduces virulence, as demonstrated in HeLa cells and *Drosophila melanogaster* models (Hittle et al., 2015; Liang et al., 2016; Wang et al., 2016), suggesting their potential to improve vaccine safety. Inactivation of both genes decreased pathogenicity, and lipid A spectra revealed presumed tetra-acylated forms, which are associated with lower TLR4/MD-2 activation, as previously reported by Hittle et al. (2015) (Hittle et al., 2015). Assays with purified LPS confirmed reduced mTLR4 activation in both mutants, correlating with a decrease in presumed penta-acylated lipid A (*m/z* 1447, 1462) and an increase in presumed tetra-acylated species. Interestingly, LPS from the Δ*htrB1* mutants elicited weaker mTLR4 activation than that from the Δ*htrB2* mutants, likely attributable to subtle structural variations, such as differences in acyl chain positioning or length, rather than differences in the relative abundance of tetra-acylated species. It should also be noted that LPS produced by *P. aeruginosa* is heterogenous, resulting in a mixture of lipid A species with potentially distinct biological activities, which represents an inherent limitation when studying lipid A structure using endogenous systems. Consequently, purified LPS preparations from these strains may differentially interact with the TLR4 signaling pathway. In this context, the observed TLR4 activity should be interpreted as the net effect of co-existing agonistic and antagonistic lipid A species within each preparation. However, lipid A composition is not the only determinant, as factors such as LPS surface exposure, membrane context, and release dynamics in whole cells may further modulate TLR4 activation, contributing to the differences observed between purified LPS and whole cells, as previously reported (Geurtsen et al., 2006). These findings partially align with prior data from *Yersinia pestis*, where tetra-acylated lipid A poorly activates TLR4 compared to hexa-acylated forms (Matsuura et al., 2010). A limitation of our analysis is the use of single-stage MALDI-TOF MS for structural assignment. While this technique provides rapid profiling of lipid modifications, structural interpretations based solely on intact mass must be considered putative, as they cannot unambiguously resolve isobaric species or internal topological arrangements without complementary orthogonal approaches such as tandem mass spectrometry (MS/MS) (Park et al., 2017). This limitation is particularly relevant for the highly abundant peak observed at *m/z* 1404. Although some studies have proposed that this ion corresponds to an atypical penta-acylated lipid A variant that emerges at this exact *m/z* in stationary-phase or mutated *P. aeruginosa* populations (Adamiak et al., 2021), it is also well documented that the ubiquitous endogenous membrane phospholipid cardiolipin (specifically the CL 68:2 isoform) generates an identical [M-H]^−^ ion at *m/z* 1404.2 (Yang et al., 2020). Such endogenous phospholipids may co-extract with lipid A during standard acid hydrolysis procedures, leading to potential isobaric interference. Therefore, unequivocal confirmation of the *m/z* 1404 assignment, as well as the fine structure of other lipid A variants, remains tentative until validated by future MS/MS fragmentation analyses.

LPS, particularly the lipid A component, plays a critical role in maintaining bacterial structural integrity. Ionic interactions between lipid A phosphate groups and Mg²^+^ ions stabilize the OM, while Van der Waals interactions among acyl chains reduce membrane fluidity and enhance stability (Molinaro et al., 2015; Simpson and Trent, 2019; Anandan and Vrielink, 2020). Overall, the lipid A-modified vaccine candidates generated in this study displayed growth profiles that closely resembled that of the parental strain. PAO1 ΔΔΔ Δ*htrB1* and PAO1 ΔΔΔ Δ*htrB2* showed a slight delay in the exponential growth. While *htrB1* deletion has not been previously associated with growth defects, *htrB2* deletion is known to delay *P. aeruginosa* growth (Hittle et al., 2015; Liang et al., 2016). Previous attempts to generate a double-deletion mutant for *htrB1* and *htrB2* were unsuccessful, likely due to impaired membrane integrity caused by reduced lipid A acylation, that may weaken Van der Waals interactions and disrupt membrane stability (Liang et al., 2016). Additionally, lipid A lacking secondary acylations may exhibit reduced binding to the flippase MsbA, hindering lipid A–Kdo translocation to the periplasm. Indeed, penta- and hexa-acylated species have greater affinity for MsbA compared to tetra-acylated lipid IV_A_ (Doerrler and Raetz, 2002). To assess environmental risk from accidental release, a water persistence study was conducted. Although ethidium bromide uptake assays have shown increased membrane permeability in *htrB1* and *htrB2* mutants (Hittle et al., 2015), no significant differences in water persistence were observed relative to the parental strain, PAO1 ΔΔΔ, indicating limited environmental persistence. Compromised membrane integrity provides a mechanistic explanation for the transcriptional cross-regulation observed among lipid A biosynthesis and modification genes. The *P. aeruginosa* cell envelope is continuously monitored by the major TCSs, primarily PhoPQ and PmrAB. Deletions of structural genes such as *htrB1* or *htrB2* drastically alter the biophysical packing of the lipid bilayer and disrupt divalent cation-mediated bridging between LPS molecules. This structural perturbation serves as an intrinsic envelope stress signal that is detected by sensor kinases such as PhoQ (Needham et al., 2013; Simpson and Trent, 2019). Upon activation, the response regulator PhoP directly orchestrates compensatory transcriptional programs to restore membrane homeostasis. This is evidenced in the PAO1 ΔΔΔ Δ*htrB1* mutant, where the loss of the secondary acyl chain triggers a marked upregulation of *pagP*. This response is consistent with a PhoPQ-driven attempt to fortify the stabilize the membrane via palmitoylation of lipid A, thereby enhancing hydrophobic interactions (Thaipisuttikul et al., 2014; Wang et al., 2021). Conversely, the down-regulation of *pagP* and *pagL* observed in the Δ*htrB2* mutant background, suggests the existence of complex negative feedback loops which likely serve to prevent deleterious extremes in OM fluidity or surface charge. Collectively, these regulatory networks enable *P. aeruginosa* to dynamically remodel its lipid A architecture not only in response to environmental stimuli, but also as a survival strategy to compensate for intrinsic genetic perturbations.

Studies on acute *P. aeruginosa* infections highlight the critical role of the humoral response in reducing pulmonary bacterial loads following IN infection. Depletion of B cells using anti-CD20 in mice reduced anti-*Pseudomonas* antibody levels and resulted in higher lung bacterial loads compared to controls (Sen-Kilic et al., 2021). Similarly, immunization with the PAO1 Δ*murI* vaccine candidate elicited serum IgG antibodies that recognized heterologous *P. aeruginosa* strains and conferred protection through passive antibody transfer in mouse models (Cabral et al., 2017, 2020). These findings highlight the importance of generating robust antibody responses through vaccination. Because *P. aeruginosa* targets mucosal surfaces, local immune activation is required for optimal protection and is supported by the concept of a common mucosal immune system (Belyakov and Ahlers, 2009). In this study, all vaccine candidates enhanced serum IgG and mucosal IgA levels specific to *P. aeruginosa*, demonstrating that lipid A modifications did not compromise the immunogenicity of the PAO1 ΔΔΔ vaccine strain. Moreover, the modified candidates conferred protection against acute pulmonary infections, showing efficacy comparable to the parental PAO1 ΔΔΔ strain. These findings represent an initial proof-of-concept in an acute pneumonia model. While this system enables robust and reproducible assessment of vaccine efficacy under stringent conditions, it does not capture the chronic and biofilm-associated nature of *P. aeruginosa* infections observed in diseases such as CF or bronchiectasis. Future studies will therefore evaluate the best vaccine candidates in chronic lung infection models to better reflect persistent human infection and further define their translational potential.

The TLR4-expressing HEK293 reporter system was deliberately selected as a highly specific model to assess the biological activity of lipid A modifications. These cells are engineered to respond exclusively through TLR4 signaling, the LPS receptor, thereby allowing direct attribution of the observed effects to changes in LPS structure, without interference from other pathogen-associated molecular patterns (PAMPs). This approach is widely used to evaluate endotoxin activity in the context of vaccine development (Arenas et al., 2020). In contrast, lung-derived epithelial cell models, such as lactate dehydrogenase (LDH) release or cytokine-based assays, capture broader cellular responses, including cytotoxicity and inflammation, and can provide a direct measure of lytic damage. Accordingly, future preclinical evaluations of live-attenuated whole-cell respiratory vaccines should incorporate lung-relevant cytotoxicity assessments together with comprehensive cytokine profiling to more accurately predict *in vivo* safety in the respiratory tract (Mahieu et al., 2024). However, these systems inherently integrate signals from multiple bacterial components (*e.g.*, lipoproteins, flagellin, peptidoglycan), making it difficult to isolate the specific contribution of LPS modifications from other PAMPs.

Importantly, our study is focused on the mechanistic validation of lipid A modification at the receptor level. Consistent with this objective, vaccine candidates engineered to express modified lipid A variants demonstrated a drastic reduction in endotoxic *in vitro* activity in mTLR4 reporter assays. Notably, this translated into significantly improved survival following high-dose IN challenge *in vivo*, supporting the rationale for these genetic modifications in mitigating lethal endotoxin shock and enhancing overall vaccine safety. Nonetheless, the persistence of transient weight loss and comparable disease severity scores at immunizing doses highlights a discrepancy between *in vitro* TLR4 reporter assays and complex *in vivo* pulmonary responses. This residual reactogenicity suggests that acute mucosal inflammation may be driven by alternative, TLR4-independent mechanisms. One possible explanation is activation of the non-canonical inflammasome pathway via cytosolic caspase-11 in mice (homologous to caspase-4/5 in humans). Binding of lipid A to caspase-11 triggers potent pyroptosis and the release of pro-inflammatory cytokines such as IL-1β and IL-18, driving acute morbidity irrespective of extracellular TLR4 signaling (Balakrishnan et al., 2018). This mechanism may explain the transient weight loss while preventing lethal TLR4-mediated systemic shock.

In summary, vaccine candidates genetically engineered to incorporate lipid A variants demonstrated reduced endotoxicity in *in vitro* assays. Nonetheless, some transient adverse effects persist at high doses in animal models. However, the inherent heterogeneity of LPS in these strains limits the extent to which specific lipid A-modifying enzymes can be individually attributed to the observed effects. Overall, these findings indicate that, although considerable progress has been made, further refinement is needed to minimize residual *in vivo* effects. Various technological approaches could be explored to enhance the vaccine candidate’s safety profile in respiratory models. These may include exploring delivery systems, formulating with adjuvants, or engineering more attenuated strains through targeted removal or regulation of virulence factors.

## Data Availability

The original contributions presented in the study are included in the article/**Supplementary Material**. Further inquiries can be directed to the corresponding authors.
