# Supplementary material for: Engineering low-endotoxin lipid A in a double auxotroph Pseudomonas aeruginosa to develop safer whole-cell vaccines
Source: Front Cell Infect Microbiol. 2026 Jun 1;16:1840122. doi: 10.3389/fcimb.2026.1840122 (PMC13265386; doi:10.3389/fcimb.2026.1840122)
Supplement: Supplementary file 1 [file DataSheet1.pdf]

Supplementary Material

Engineering low-endotoxin lipid A in a double auxotroph *Pseudomonas aeruginosa* to develop safer whole-cell vaccines

Víctor Fuentes-Valverde[1,2†], Patricia García[1,2], Ana Candela[1], Rebeca Santamarina-Fernández[1], José Avendaño-Ortiz[2,3], Emma Martínez-Alonso[4,5], Marina Oviaño[1,2], Rafael Cantón[2,3], Jesús Arenas[6], Miriam Moscoso[1,2*‡], Germán Bou[1,2,7*‡]

[1]Servicio de Microbiología Clínica and Grupo de Investigación en Microbiología, Instituto de Investigación Biomédica de A Coruña (INIBIC), Complexo Hospitalario Universitario de A Coruña (CHUAC), SERGAS, Universidade da Coruña, A Coruña, Spain

[2]Centro de Investigación Biomédica en Red de Enfermedades Infecciosas (CIBERINFEC), Instituto de Salud Carlos III, Madrid, Spain

[3]Servicio de Microbiología, Hospital Universitario Ramón y Cajal and Instituto Ramón y Cajal de Investigación Sanitaria (IRYCIS), Madrid, Spain

[4]Department of Research, Hospital Universitario Ramón y Cajal, Instituto Ramón y Cajal de Investigación Sanitaria (IRYCIS), Madrid, Spain

[5]Proteomics Unit, Hospital Universitario Ramón y Cajal, Instituto Ramón y Cajal de Investigación Sanitaria (IRYCIS), Madrid, Spain

[6]Unit of Microbiology and Immunology, Faculty of Veterinary, University of Zaragoza, Agroalimentary Institute of Aragon, IA2. II2, Zaragoza, Spain.

[7]Departamento de Fisioterapia, Medicina e Ciencias Biomédicas, Universidade da Coruña, A Coruña, Spain

[†]Present address: Área de Medicamentos Biológicos, Agencia Española de Medicamentos y Productos Sanitarios (AEMPS), Madrid, Spain

*To whom correspondence may be addressed: Germán Bou, German.Bou.Arevalo@sergas.es or Miriam Moscoso, Mirian.Moscoso.Naya@sergas.es.

[‡]These authors have contributed equally to this work.

1 Supplementary Figures and Tables

1.1 Supplementary Figures

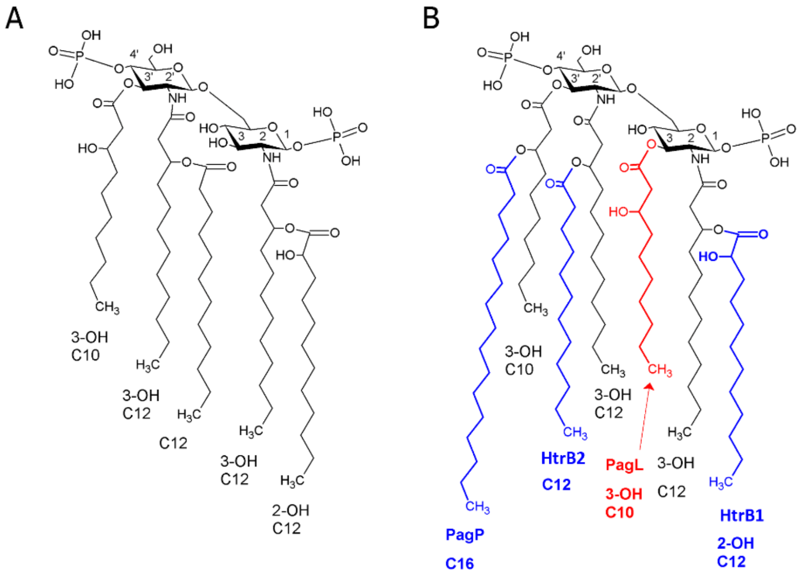

Supplementary Figure 1. Structures of lipid A molecules synthesized by *P aeruginosa*. **A.** Structure of the bis-phosphorylated, penta-acylated lipid A from *P. aeruginosa*, consisting of a diglucosamine backbone, one 3-OH-C10 and two 3-OH-C12 as primary fatty acids, with 2-OH-C12 and C12 as secondary acylations, and two phosphate groups. **B.** Modifications to lipid A structure by key enzymes: PagP, adding a secondary palmitate chain (C16) at the 3’ position; HtrB2 (PA3242), introducing a secondary laurate group (C12) at the 2’ position; PagL, removing 3-OH-C10 from position 3; and HtrB1 (PA0011), adding 2-hydroxylaurate at position 2. Removed groups and enzymes are shown in red; added groups and responsible enzymes are in blue. Images created with ChemSketch software.

Supplementary Figure 2. Analysis of deletion mutants with modified LPS derived from the PAO1 ΔΔΔ vaccine strain. **A.** Schematic representation of the genetic regions flanking genes *htrB1* (PA0011), *htrB2* (PA3242), *pagP* (PA1343) and *pagL* (PA4661). **B.** PCR amplification of the *murI*, *alr*, *dadX*, *htrB1*, *htrB2*, *pagP* and *pagL* gene regions from PAO1 and PAO1 ΔΔΔ and the deletion mutants using specific primers. Electrophoresis was performed on 0.9% agarose gels at 100 V for 30–40 min. The molecular weight marker (M) used was GeneRuler 1 kb Plus DNA Ladder (Thermo Scientific, SM1331). Lanes: wt, PAO1 (wild-type phenotype); 1, PAO1 ΔΔΔ; 2, PAO1 ΔΔΔ Δ*htrB1*; 3, PAO1 ΔΔΔ Δ*htrB2*; 4, PAO1 ΔΔΔ Δ*pagP*; 5, PAO1 ΔΔΔ Δ*htrB1* Δ*pagP*; 6, PAO1 ΔΔΔ Δ*htrB2* Δ*pagP*; 7, PAO1 ΔΔΔ Δ*pagL*.

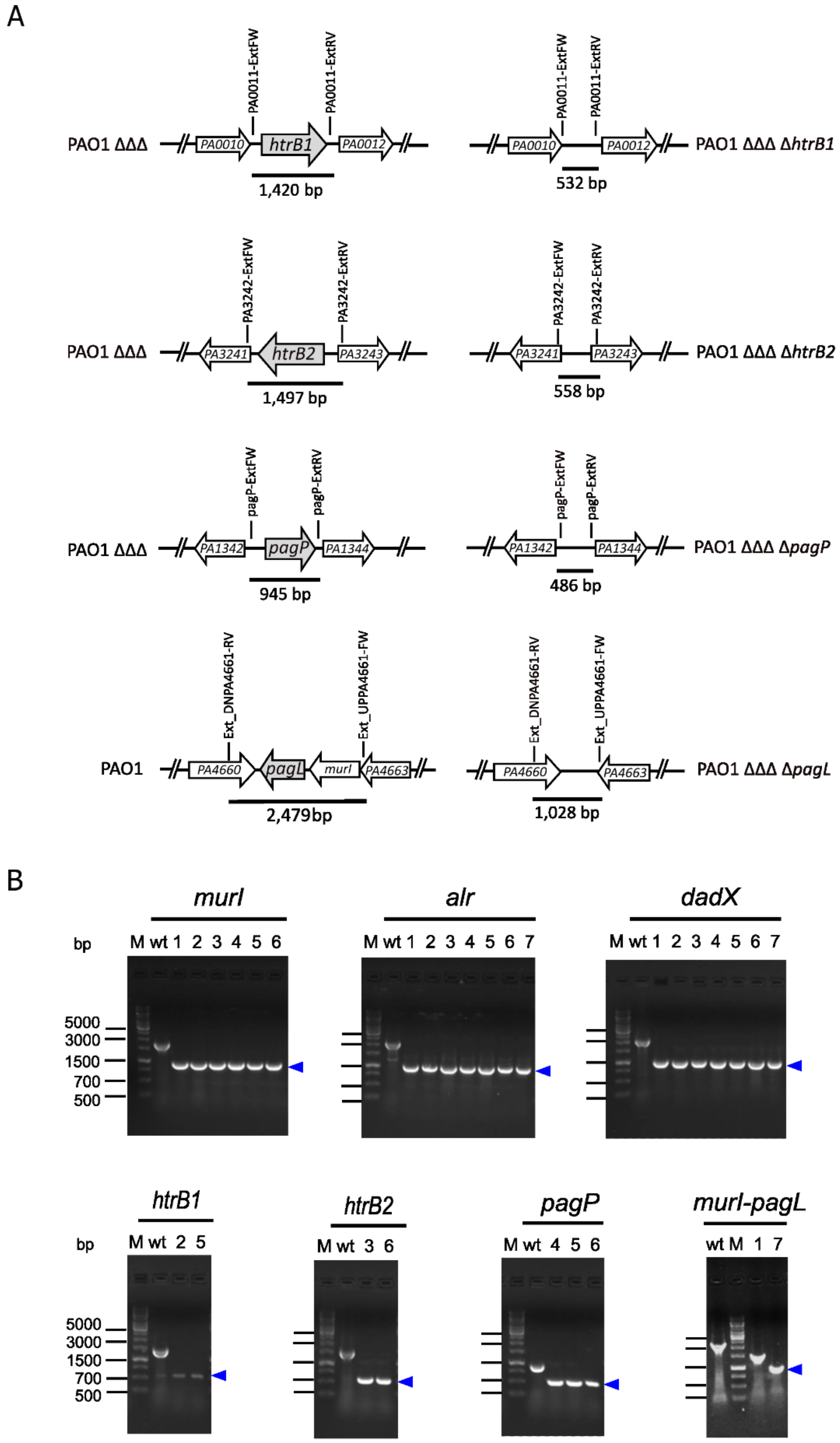
A
PAO1 ΔΔΔ
PA0011-ExtFW
PA0011-ExtRV
PA0010
htrB1
PA0012
1,420 bp
PAO1 ΔΔΔ ΔhtrB1
532 bp
PA3242-ExtFW
PA3242-ExtRV
PA3241
htrB2
PA3243
1,497 bp
PAO1 ΔΔΔ ΔhtrB2
558 bp
pagP-ExtFW
pagP-ExtRV
PA1342
pagP
PA1344
945 bp
PAO1 ΔΔΔ ΔpagP
486 bp
PAO1
Ext_DNPA4661-RV
Ext_UPPA4661-FW
PA4660
pagL
murI
PA4663
2,479bp
PAO1 ΔΔΔ ΔpagL
1,028 bp
B
murI
alr
dadX
bp
M wt 1 2 3 4 5 6
M wt 1 2 3 4 5 6 7
5000
3000
1500
700
500
htrB1
htrB2
pagP
murI-pagL
M wt 2 5
M wt 3 6
M wt 4 5 6
wt M 1 7

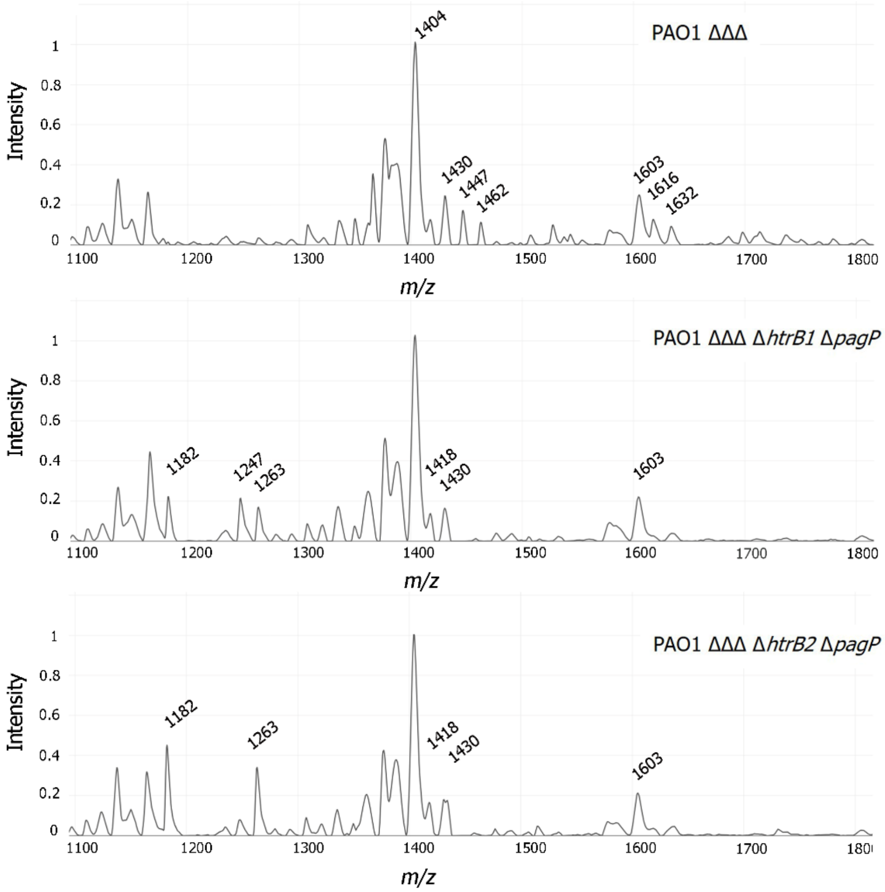

Supplementary Figure 3. Structural analysis of lipid A from *P. aeruginosa* double acyltransferase-deficient mutants. MALDI-TOF MS spectra in negative-ion mode for the following strains: PAO1 ΔΔΔ, PAO1 ΔΔΔ Δ*htrB1* Δ*pagP*, PAO1 ΔΔΔ Δ*htrB2* Δ*pagP*. The displayed *m/z* range (1100-1800) shows the main lipid A ion species. Intensity is expressed as relative intensity, normalized to the mass peak at *m/z* 1404.

1.2 Supplementary Tables

Supplementary Table 1. List of oligonucleotides used in this study.

Identification	Sequence* (5' to 3')	Amplicon size (bp)
**Verification of gene deletions of PAO1 ΔΔΔ strain**		
*alr*_EXT F	GATCATGATCGACTACCT	2069 (wt)<br>992 (Δ*alr*)
*alr*_EXT R	GATGGAGTTCGCCGAAAG	
*dadX*_EXT F	CGCAGATCAGTACCGAAG	2101 (wt)<br>1051 (Δ*dadX*)
*dadX*_EXT R	CGTGGTTGAGCATTTCCT	
PA4662_EXTFW	GTATCGGCAAGGTGGAGT	1741 (wt)<br>943 (Δ*murI*)
PA4662_EXTRV	GAATGGCTTGATCGAGTC	
**Generation of the mutant derivatives of PAO1 ΔΔΔ strain with modified LPS**		
UP_PA0011 (FW) (HindIII)	CCC**AAGCTT**CGCGGAAGTATACCCGATTCC	872
UP_PA0011 (RV) (NotI)	CCC**GCGGCCGC**CTCCACGAAACACTCCTCGCT	
DOWN_PA0011 (FW) (NotI)	CCC**GCGGCCGC**TGGTATTGAGGAAAGACG	826
DOWN_PA0011 (RV) (XbaI)	CCC**TCTAGA**GGGCAGGGCCCTTGC	
PA0011-EXT-FW	TGCTACGCCTTCATGCAG	1420 (wt)<br>532 (Δ*htrB1*)
PA0011-EXT-RV	ACTCGCACGACTTCCATC	
UP_PA3242 (FW) (HindIII)	CCC**AAGCTT**GGGGTCTTCACCACC	854
UP_PA3242 (RV) (NotI)	CCC**GCGGCCGC**GCGATCCATGGACGG	
DOWN_PA3242 (FW) (NotI)	CCC**GCGGCCGC**CGCTGAGGACGGGCATT	704
DOWN_PA3242 (RV) (XbaI)	CCC**TCTAGA**GCCCAGGCGCCGATG	
PA3242-EXT-FW	TTGTCGAGGGCCATGACCAG	1497 (wt)<br>558 (Δ*htrB2*)
PA3242-EXT-RV	ACCGACGATGACCGGGAAAG	
UP_PA1343 (FW) (HindIII)	CCC**AAGCTT**GCCGATCGGCTTGCCCGG	739

UP_PA1343 (RV) (NotI)	CCC**GCGGCCGC**GCGCATGGGGACTCCAGG	
DOWN_PA1343 (FW) (NotI)	CCC**GCGGCCGC**CGTCTCTGAGGCCAG	648
DOWN_PA1343 (RV) (XbaI)	CCC**TCTAGA**CGCCGATTCCGAAGA	
pagP-EXT-FW	TCGTGCTTGCCGAACAGTG	945 (wt)<br>486 (Δ*pagP*)
pagP-EXT-RV	TGGAACCGCTGACGATTGC	
UP_PA4661_FW (HindIII)	CCC**AAGCTT**GACGGCTGCGGCTGCGGC	1179
UP_PA4661_RV (NotI)	CCC**GCGGCCGC**TGAAACGTCCTTTTCGAAAGTTG	
UP_PA4662_F II (HindIII)	CCC**AAGCTT**GGCAATCCGCCGTATATC	1032
UP_PA4662_RV (NotI)	CCC**GCGGCCGC**GGCGTTGCCCGCAGACGG	
DOWN_PA4661_FW (NotI)	CCC**GCGGCCGC**TCGGCACGGGCGAAAAAAGC	699
DOWN_PA4661_RV (XbaI)	CCC**TCTAGA**GCACATCCTGGTCGGCTACC	
PA4661_EXT_FW	CTGGCGCCCATCCTCGAAC	1510 (wt)<br>988 (Δ*pagL*)
PA4661_EXT_RV	CTGTTCAATCCGCTCTCGCAG	
EXT_UP PA4661_FW	GGTGGACGCCCTCGGCACG	2479 (wt)<br>1681 (ΔΔΔ)<br>1028 (ΔΔΔ Δ*pagL*)
EXT_DN PA4661_RV	CGGCAGGCGAAGAGGTAGCG	
**Universal primers**		
pEx18 universal FW	GGCTCGTATGTTGTGTGGAATTGTG	205 (without insert)
pEx18 universal RV	GGATGTGCTGCAAGGCGATTAAG	
UpKn	CCCTGGATTTCACTGATGAG	569 (without insert)
RpKn	CATATCACAACGTGCGTGGA	

*Restriction sites introduced into the sequence for cloning are indicated in bold. bp: Base pairs. MCS: the Multiple Cloning Site. wt: Wild-type phenotype.

Supplementary Table 2. Specific primers and UPL probes used for RT-PCR reactions.

Name	Sequence (5' to 3')	UPL probes*
htrB1#46_left	GCGGACATGTACGACAAGGA	46
htrB1#46_right	GGCTCGTACATACTTCGCCA	
htrB2#49_left	GGCATCGCCTTCTTCGAGAT	49
htrB2#49_right	TCTATATGGGCAAGACGGGC	
pagP#26_left	ATCTCATCCTCAGCCTGCTG	26
pagP#26_right	CTGCAGGTACCAGAAGTCGC	
pagL#124_left	CCTGAACTTCGAAGACCGCA	124
pagL#124_right	AATAGTGGATCGCCCGAACG	
eptA#156_left	GCCGGTACCACCTACTTCAT	156
eptA#156_right	GGTTGGTCTGCATGACGTTG	
rpoS#63_left	AAGAAGGGCCGGAGTTTGAC	63
rpoS#63_right	ACGACTCGTCCAGCATGATG	

* UPL probes, TaqMan probes from the Universal Probe Library (UPL) by Roche.

Supplementary Table 3. Vaccine doses (in CFU) of different strains administered via the intranasal route.

Strain	Vaccine dose 1 (CFU)	Vaccine dose 2 (CFU)
PAO1 ΔΔΔ	4.00×10^{8}	3.54×10^{8}
PAO1 ΔΔΔ Δ*htrB1*	3.96×10^{8}	3.95×10^{8}
PAO1 ΔΔΔ Δ*htrB2*	3.07×10^{8}	3.03×10^{8}
PAO1 ΔΔΔ Δ*pagP*	3.56×10^{8}	2.99×10^{8}
